# 2-D Stationary Wavelet Transform and 2-D Dual-Tree DWT for MRI Denoising

**DOI:** 10.2174/0115734056365765250630140748

**Published:** 2025-07-07

**Authors:** Mourad Talbi, Brahim Nasraoui, Arij Alfaidi

**Affiliations:** 1 Laboratory LMEEVED, Center of Biotechnogy of Borj Cédria, Hammam-Lif, Tunisia; 2 Department of Computer Sciences, University College of Duba, University of Tabuk, Tabuk, Saudi Arabia

**Keywords:** Denoising techniques, Thresholding, MR Image, SWT-2D, 2D Dual-Tree DWT, Feature Similarity

## Abstract

**Introduction:**

The noise emergence in the digital image can occur throughout image acquisition, transmission, and processing steps. Consequently, eliminating the noise from the digital image is required before further processing. This study aims to denoise noisy images (including Magnetic Resonance Images (**MRIs**)) by employing our proposed image denoising approach.

**Methods:**

This proposed approach is based on the Stationary Wavelet Transform (***SWT* 2-*D***) and the **2 - *D*** Dual-Tree Discrete Wavelet Transform (**DWT**). The first step of this approach consists of applying the **2 - *D*** Dual-Tree **DWT** to the noisy image to obtain noisy wavelet coefficients. The second step of this approach consists of denoising each of these coefficients by applying an *SWT* 2-*D* based denoising technique. The denoised image is finally obtained by applying the inverse of the 2-D Dual-Tree **DWT** to the denoised coefficients obtained in the second step. The proposed image denoising approach is evaluated by comparing it to four denoising techniques existing in literature. The latters are the image denoising technique based on thresholding in the **SWT-2D** domain, the image denoising technique based on deep neural network, the image denoising technique based on soft thresholding in the domain of 2-D Dual-Tree DWT, and Non-local Means Filter.

**Results:**

The proposed denoising approach, and the other four techniques previously mentioned, are applied to a number of noisy grey scale images and noisy Magnetic Resonance Images (***MRIs***) and the obtained results are in terms of ***PSNR*** (Peak Signal to Noise Ratio), ***SSIM*** (Structural Similarity), ***NMSE*** (Normalized Mean Square Error) and Feature Similarity (***FSIM***). These results show that the proposed image denoising approach outperforms the other denoising techniques applied for our evaluation.

**Discussion:**

In comparison with the four denoising techniques applied for our evaluation, the proposed approach permits to obtain highest values of ***PSNR***, ***SSIM*** and ***FSIM*** and the lowest values of ***NMSE***. Moreover, in cases where the noise level **σ = 10** or **σ = 20**, this approach permits the elimination of the noise from the noisy images and introduces slight distortions on the details of the original images. However, in case where **σ = 30** or **σ = 40**, this approach eliminates a great part of the noise and introduces some distortions on the original images.

**Conclusion:**

The performance of this approach is proven by comparing it to four image denoising techniques existing in literature. These techniques are the denoising technique based on thresholding in the SWT-2D domain, the image denoising technique based on a deep neural network, the image denoising technique based on soft thresholding in the domain of ***2 - D*** Dual-Tree ***DWT*** and the Non-local Means Filter. All these denoising techniques, including our approach, are applied to a number of noisy grey scale images and noisy ***MRIs***, and the obtained results are in terms of ***PSNR*** (Peak Signal to Noise Ratio), ***SSIM***(Structural Similarity), ***NMSE*** (Normalized Mean Square Error) and ***FSIM*** (Feature Similarity). These results show that this proposed approach outperforms the four denoising techniques applied for our evaluation.

## 1 NTRODUCTION

The presence of noise in the digital image can occur throughout image acquisition, transmission, and processing stages [1, 2]. Additive Gaussian White Noise (AGWN) is a kind of noise that usually occurs in various systems [3]. Consequently, eliminating this kind of noise from a noisy image is required prior to further processing such as texture analysis, feature extraction, and segmentation [4]. Conserving important features of an image, such as edges and textures, is one of the principal issues faced throughout the denoising procedure [5]. Though due to the fact that the noise, texture, and edge are high-frequency components, it is not easy to discriminate them in the denoising procedure, and the denoised image could ineluctably lose some important details [6]. Many approaches were introduced in the literature and among them, we can mention wavelet transforms and non-local means filters [7]. In the thresholding in the wavelet transform domain, the noisy image is decomposed into the low and high frequency sub-bands. Those frequency sub-bands were thresholded. This thresholding is quite efficient when applied to the high-frequency sub-bands. However, it fails in the case where applied to the low-frequency sub-band [8]. The DWT has been extensively employed. However, this transform has three drawbacks, which are shift invariance, aliasing, and lack of poor directionality [9]. Besides, DWT provides the advantages of adaptation and smoothness, as Donoho and Coifman [10] suggested, DWT exhibits visual artefacts recognized as Gibbs phenomena in the discontinuities [1]. For addressing this problem, the translation invariant denoising approach named cycle spinning was applied to eliminate such artefacts. Meanwhile, DT − CWT is a technique proposed for solving the principal problems of DWT, which was combined with Non-Local Means (NLM) [11-13]. Two well-known wavelet transforms, DWT and DT-CWT, were applied sequentially. The low-frequency sub-band is only denoised employing NLM filtering. Since the high-frequency sub-bands contained noises, these were denoised employing hard thresholding with cycle spinning. The inverse of DWT is applied to the modified sub-bands for reconstructing the denoised image [1].

Conventional image denoising techniques not only cancel the noise corrupting an image, but also result in the loss in this image. They cannot insure the clean cancellation of noise information while conserving the true signal of this image. For addressing the aforementioned issues, an image denoising technique that combines an improved threshold function and wavelet transform was introduced [14].

In contrast to conventional threshold functions, the ameliorated threshold function is a continuous function which can avoid the pseudo Gibbs effect after image denoising and ameliorate the image quality. During the process, the output image of the finite ridge wave transform is first combined with the wavelet transform to ameliorate the denoising performance. After that, an ameliorated threshold function is proposed for enhancing the reconstructed image quality [14]. Our proposed image denoising technique [15, 16] consists of applying an *SWT* 2-*D* based denoising approach [17] to each of the noisy wavelet coefficients, named *Wb*{*j*}{s},1 ≤ *j* ≤ 2, 1 ≤ *s* ≤ 3. These coefficients are obtained by applying the 2 – D Dual-Tree DWT to the noisy image. The denoising approach [17] based on *SWT* 2-*D* consists of applying the soft thresholding of the noisy wavelet coefficients obtained from the noisy image decomposition employing the *SWT* 2-*D*. For this decomposition, the Daubechies four, ‘db4’, is employed as the mother wavelet, and the level of this decomposition is equal to five. The novelty of our proposed image denoising approach [15, 16] lies in applying the thresholding approach in the *SWT* 2-*D* domain, not to the whole noisy image but to each of the coefficients, *Wb*{*j*}{s},1 ≤ *j* ≤ 2, 1 ≤ *s* ≤ 3, for denoising it. This results in denoised coefficients, named *Wd*{*j*}{s},1 ≤ *j* ≤ 2, 1 ≤ *s* ≤ 3. The denoised image is finally obtained by applying the inverse of the 2 – D Dual-Tree DWT to *Wd*{*j*}{s},1 ≤ *j* ≤ 2, 1 ≤ *s* ≤ 3. The remaining of this paper is prearranged as follows: in section 2, Materials and Methods are described. In section 3, the obtained Results and Discussion are presented. Lastly, Conclusion is presented in section 4.

## 2 MATERIALS AND METHODS

### 2.1 A *SWT* 2-*D* based Image Denoising Approach

A denoising approach based on thresholding in the *SWT* 2-*D* domain [17] is applied in our denoising system [15, 16]. This proposed approach [17] is summarized by the block diagram illustrated in Fig. (**1**).

According to Fig. (**1**), the *SWT* 2-*D* is at the first step applied to the noisy image (denoted as *I_b_*) to have noisy stationary wavelet coefficients. The latters are secondly denoised by applying the soft thresholding and finally the inverse of (*SWT*^-1^ 2-*D*) is applied to the thresholded coefficients (obtained at the second step) to have the denoised image (denoted as *I_d_*). As illustrated in Fig. (**1**), for applying the *SWT* 2-*D*, the used mother wavelet is the Daubechies four, ‘*db*4’ and the decomposition level is equal to 5.

## 3 THE PROPOSED IMAGE DENOISING TECHNIQUE [15, 16]

In this section, we present our proposed denoising approach [15, 16]. It is summarized by the block diagram illustrated in Fig. (**2**).

**Fig. (1):**
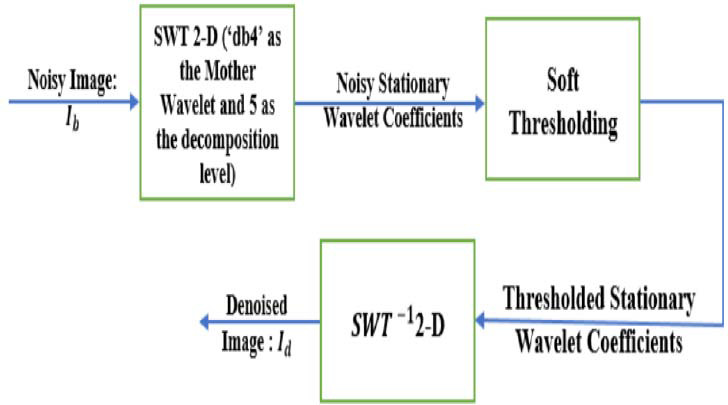
The block diagram of a denoising approach based on thresholding in the *SWT* 2-*D* domain [17].

As illustrated in Fig. (**2**, the 2-D dual-Tree DWT is firstly applied to the noisy image (named *I_b_*) to have noisy wavelet coefficients, *Wb*{*j*}{s},1 ≤ *j* ≤ 2, 1 ≤ *s* ≤ 3 (*Wb*{1}{1}, *Wb*{1}{2}, *Wb*{1}{3}, *Wb*{2}{1}, *Wb*{2}{2}, and *Wb*{2}{3}). Each of these coefficients is secondly considered as a noisy image and is denoised by applying the denoising approach based on thresholding in the *SWT* − 2*D* domain [17] (Fig. **1**). The denoised wavelet coefficients obtained in this step, are named *Wd*{*j*}{s},1 ≤ *j* ≤ 2, 1 ≤ *s* ≤ 3 (*Wd*{1}{1}, *Wd*{1}{2}, *Wd*{1}{3}, *Wd*{2}{1}, *Wd*{2}{2}, and *Wd*{2}{3}). In the last step of the proposed approach [15, 16], the inverse of the 2 − D dual − Tree DWT is applied to the coefficients, *Wd*{*j*}{s},1 ≤ *j* ≤ 2, 1 ≤ *s* ≤ 3 for obtaining the denoised image (named *I_d_*). Also for applying the denoising approach based on thresholding in the *SWT* 2-*D* domain [17] (Fig. **1**), the *db*4 is also employed as the mother wavelet. Furthermore, the decomposition level is chosen to be equal to 5. These choices were justified [16]. In a study [15, 16], the proposed image denoising technique was applied to grey-scale images and in this work, it is applied to both grey-scale and Magnectic Resonance (MR) Images in order to prove its performance.

## 4 RESULTS AND DISCUSSION

In this work, we evaluate the proposed image denoising approach [15, 16] by comparing it to three other denoising techniques introduced in the literature. These three techniques are the image denoising technique based on thresholding in the *SWT* 2 – *D* domain [17], the image denoising technique based on soft thresholding in the domain of 2-D Dual-Tree DWT [18] and the image denoising technique based on a deep neural network [19]. All the denoising techniques, including the proposed image denoising approach [15, 16], are applied for denoising a number of gray scale images corrupted by an additive Gaussian white noise with different values of standard deviation, σ (10, 20, 30 and 40), and the obtained results are in terms of PSNR (Peak Signal to Noise Ratio) and SSIM (Structural Similarity). These results are listed in Table **1**.

The best results listed in Table **1** are highlighted in **purple**, which show that the proposed image denoising approach outperforms the three other denoising techniques applied for our evaluation [17-19].

**Fig. (2):**
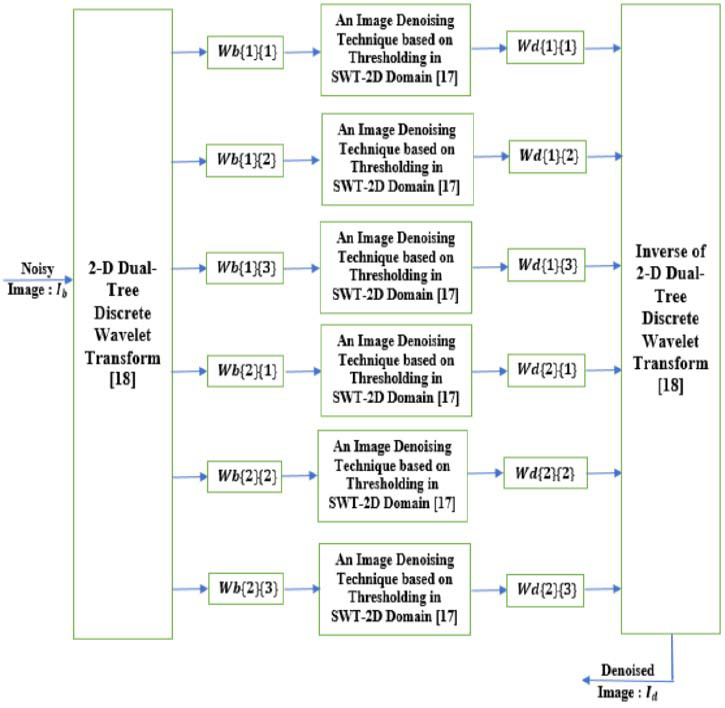
The block diagram of the proposed image denoising approach [15, 16].

In this part, some results are listed for diverse image denoising examples. These examples are obtained by applying the proposed denoising technique (Fig. **2**) [15, 16]. These results are also in terms of PSNR and SSIM [15, 16]. These examples are illustrated in Figs. (**3**-**5**).

**Fig. (3):**
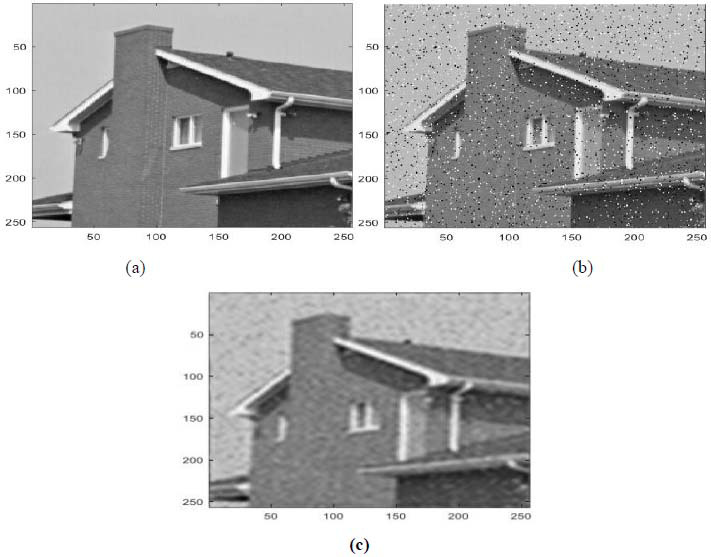
A first example of image denoising: (**a**) The clean image ('house.tif'), (**b**) The noisy image (The clean image ('house.tif') corrupted by an additive salt and peppers noise with PSNR = 66.5736 dB, SSIM = 0.9997), (**c**) The denoised image obtained *via* the application of the approach introduced [15, 16] (*PSNR* = 74.7058 *dB, SSIM =* 0.9999).

**Fig. (5):**
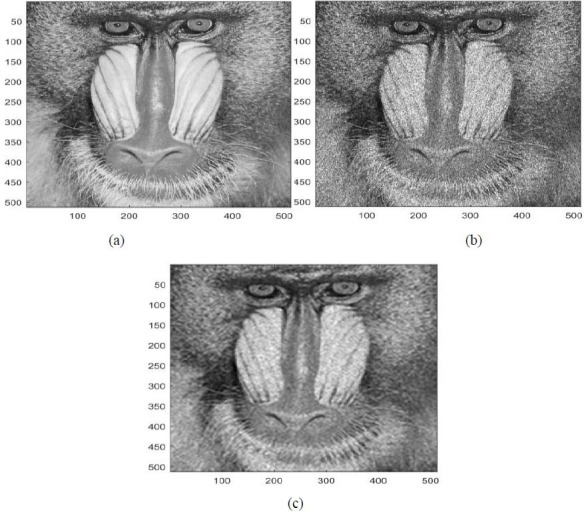
A third example of image denoising: (**a**) The clean image ('mandrill.tif'), (**b**) The noisy image (Clean image ('mandrill.tif') corrupted by adding Poisson noise with PSNR1 = 63.9702 dB, SSIM1 = 0.9994), (**c**) Denoised image obtained *via* the application of the approach introduced [15, 16] (*PSNR* = 69.1995 dB, *SSIM* = 0.9998).

Figs. (**3**-**5**) and the results (in terms of PSNR and SSIM) obtained by applying the image denoising approach introduced [15, 16] prove the performance of this approach [15, 16]. In fact, this approach permits considerable reduction of the noise and conserves the different details of the original images, precisely in cases where the *PSNR*1 (*PSNR* before denoising) is higher (Figs. **3** and **5**). Furthermore, this proposed technique permits considerable improvement in the PSNR to obtain SSIM values near 1 (Figs. **3** and **5**).

We have also applied the denoising approach introduced [15, 16] to a number of Magnetic Resonance (MR) images. Figs. (**6**-**9**) illustrate four examples of MR image denoising by applying this denoising technique [15, 16].

Figs. (**6**-**9**) and the results (in terms of PSNR and SSIM) obtained by applying the image denoising technique introduced [15, 16] to four MR images corrupted by Additive Gaussian White noise, show the performance of this approach [15, 16]. In fact, when the noise level, *σ*, is low (in cases where *σ* = 10 and *σ* = 20, as illustrated in Figs. (**6** and **9**), this technique permits the elimination of the noise from the noisy image and preserves the different details of the original image. However, when *σ* = 30, *σ* = 40 (as illustrated in Figs. (**7** and **8**), this technique permits the elimination of a great amount of noise and introduces some distortions on the original images.

In this section, we also present a comparative study between the proposed image denoising technique [15, 16] and the non-local-means filter [20]. This comparative study is performed by computing PSNR, SSIM, NMSE (Normalized Mean Square Error) and Feature Similarity (FSIM) and the obtained results are listed as follows:

According to the results listed in Tables **2**-**13**, the best results are highlighted in purple and are approximately obtained by the proposed image denoising technique [15, 16]. Consequently, this technique [15, 16] outperforms the Non-Local Means Filter [20]. Figs. (**10**-**21**) show the performance of the proposed image denoising technique [15, 16]. In fact, in cases where σ = 10 or σ = 20, this proposed technique [15, 16] permits the elimination of the noise from the noisy images and introduces slight distortions on the different details of the original images. However, in cases where σ = 30 or σ = 40, this approach eliminates a great part of the noise and introduces some distortions on the original images.

## CONCLUSION

This study aimed to denoise noisy images by employing a denoising approach based on the *SWT* 2 - D and the 2 −D Dual − Tree DWT. This approach lies in the first step by applying the 2 − D Dual − Tree DWT to the noisy image to obtain noisy wavelet coefficients. The second step of this technique consists of denoising each of these coefficients by applying an SWT-2D based denoising technique. The denoised image is finally obtained by applying the inverse of the 2 − D Dual − Tree DWT to the denoised coefficients obtained in the second step. The performance of this technique is proven by its comparison to three other techniques, which are the soft thresholding in the *SWT* 2 - D domain, the soft thresholding in the 2 − D Dual − Tree DWT domain and the denoising technique based on a deep neural network. All these techniques, including the proposed denoising technique [15, 16], are applied to a number of noisy images, and the obtained results are in terms of PSNR and SSIM. These results show that this approach [15, 16] outperforms the three other techniques [17-19]. We have also applied this proposed approach [15, 16] to a number of noisy MR images, and the results are obtained by calculating the PSNR and SSIM. These results prove the performance of the proposed approach [15, 16]. In fact, when the noise level *σ* = 10 or *σ* = 20, this approach permits the cancellation of the noise from the noisy images and preserves the different details of the original images. Whereas, when *σ* = 30 or *σ* = 40, this technique permits the elimination of a great amount of noise and introduces some distortions on the original images. We also made a comparative study between the proposed approach [15, 16] and the non-local-means filter [20]. This study is performed by computing PSNR, SSIM, NMSE (Normalized Mean Square Error) and Feature Similarity (FSIM) and the obtained results show that the proposed approach [15, 16] outperforms the Non Local Means Filter [20].

## AUTHORS’ CONTRIBUTIONS

It is hereby acknowledged that all authors have accepted responsibility for the manuscript's content and consented to its submission. They have meticulously reviewed all results and unanimously approved the final version of the manuscript.

## Figures and Tables

**Fig. (4):**
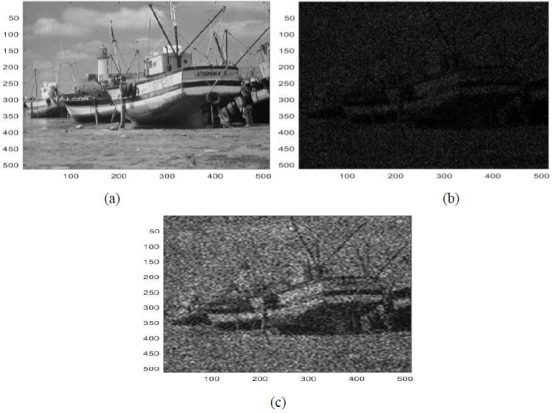
A second example of image denoising: (**a**) The clean image ('boat.tif'), (**b**) The noisy image (Clean image ('boat.tif') corrupted by adding Poisson noise with PSNR = 51.0684 dB, SSIM = 0.9893), (**c**) The denoised image obtained *via* the application of the image denoising approach introduced [15, 16] (*PSNR* = 63.6801 dB, *SSIM* = 0.9980).

**Fig. (6):**
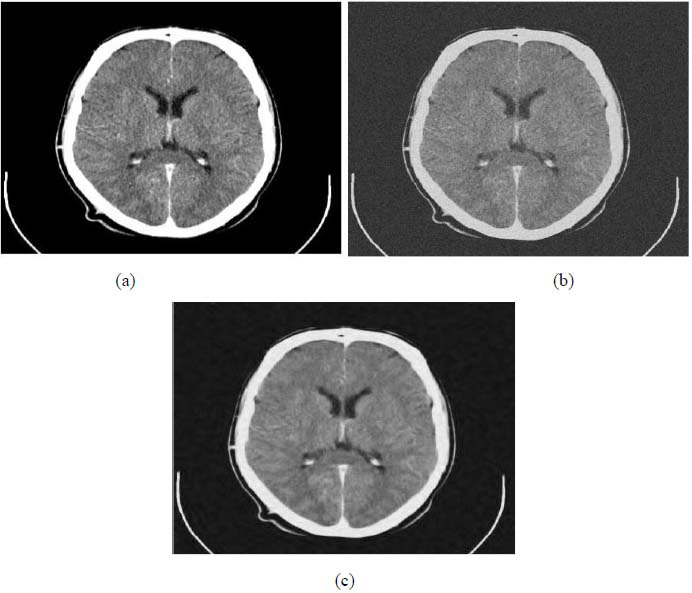
First example of image denoising: (**a**) The clean Image ('0_fNRjMcFmTjiC3c1K.png'), (**b**) the noisy Image (Clean image ('0_fNRjMcFmTjiC3c1K.png') degraded by adding a Gaussian White noise with the noise level, *σ* = 20, PSNR1 = 22.1376 dB, and *SSIM*1 = 0.3229, (**c**) Denoised image obtained *via* the application of the approach introduced [15, 16] (PSNR2 = 28.7066 dB, SSIM2 = 0.6357).

**Fig. (7):**
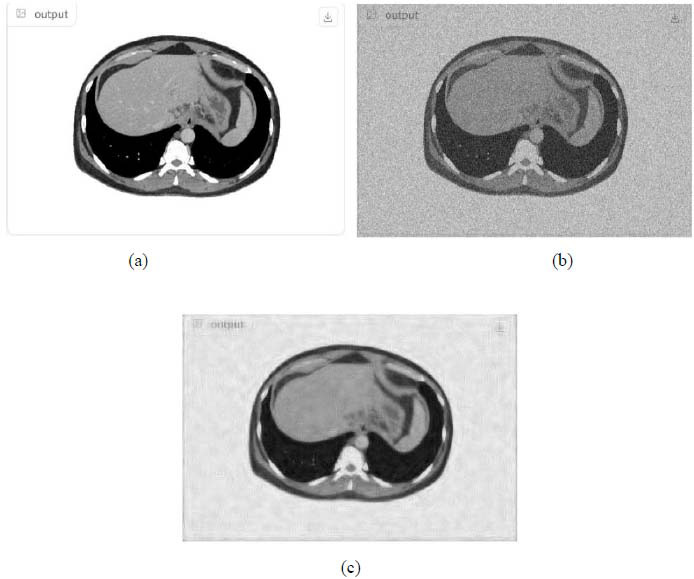
Second example of image denoising: (**a**) The clean Image ('232001710-094d1257-f1fa-479d-98be-725c8673e80b.png'), (**b**) The noisy Image (Clean image ('232001710-094d1257-f1fa-479d-98be-725c8673e80b.png') corrupted by adding a Gaussian White noise with the noise level, *σ* = 30, PSNR1 = 18.5782 dB, and SSIM1 = 0.2169, (**c**) Denoised image obtained *via* the application of the denoising scheme introduced [15, 16] (PSNR2 = 26.7592 dB, SSIM2 = 0.6211).

**Fig. (8):**
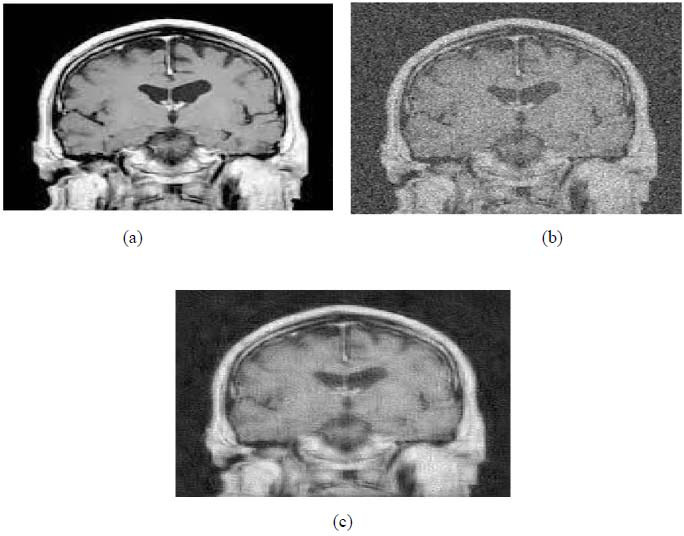
Third example of image denoising: (**a**) The clean image ('download.jpg'), (**b**) The noisy image (Clean image ('download.jpg') corrupted by adding a Gaussian White noise with the noise level, *σ* = 40, PSNR1 = 16.0831dB, and *SSIM*1 = 0.4106, (**c**) Denoised image obtained *via* the application of the approach introduced [15, 16] (PSNR2 = 22.0748 dB, SSIM2 = 0.5760).

**Fig. (9):**
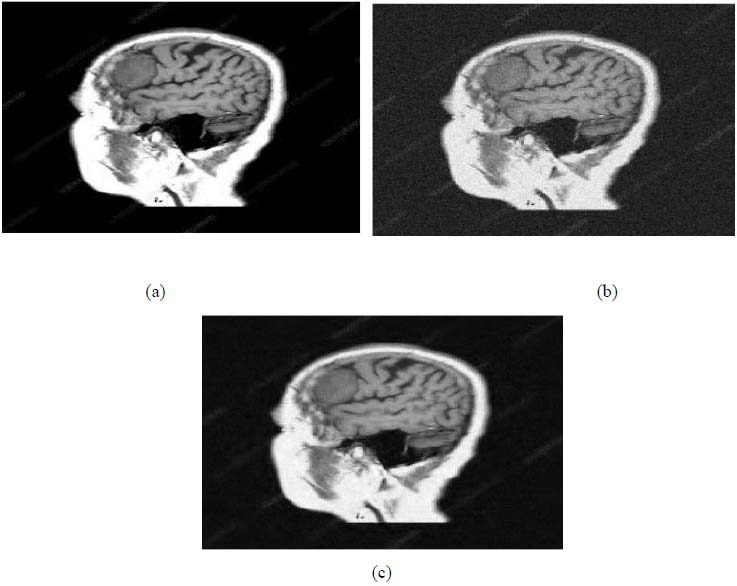
Fourth example of image denoising: (**a**) The clean image ('800wm.jpg’), (**b**) The noisy image (Clean image ('800wm.jpg’) corrupted by adding a Gaussian White noise with the noise level, *σ* = 10, PSNR1 = 28.1511dB, and SSIM1 = 0.5736, (**c**) Denoised image obtained *via* the application of the approach introduced [15, 16] (PSNR2 = 33.0660 dB, SSIM2 = 0.8152).

**Fig. (10):**
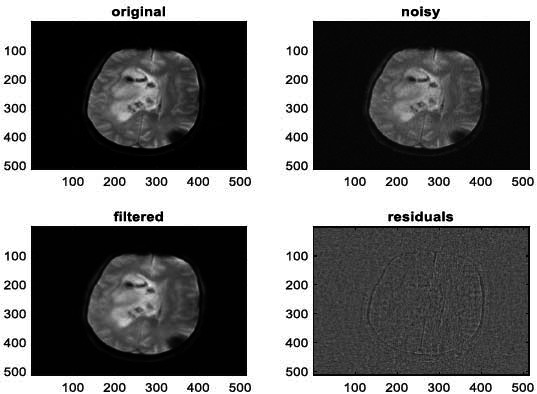
The first example of MRI Denoising by applying the proposed image denoising technique.

**Fig. (11):**
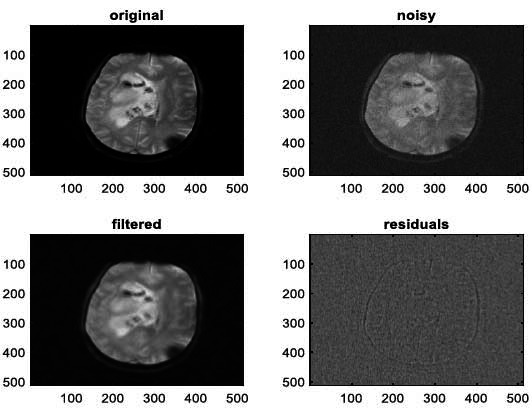
The second example of MRI denoising by applying the proposed image denoising approach.

**Fig. (12):**
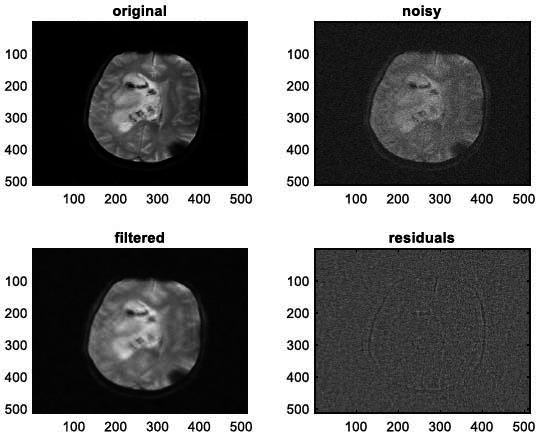
The third example of MRI denoising by applying the proposed image denoising technique.

**Fig. (13):**
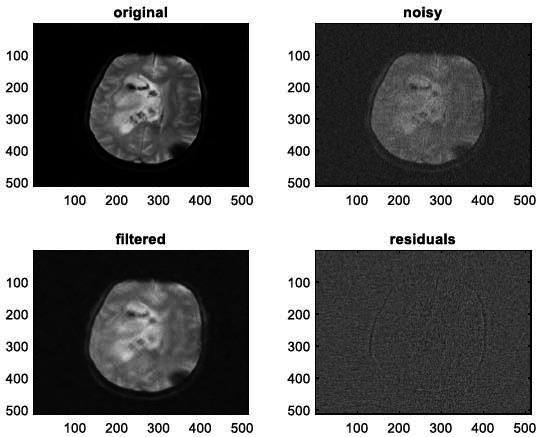
The fourth example of MRI denoising by applying the proposed image denoising technique.

**Fig. (14):**
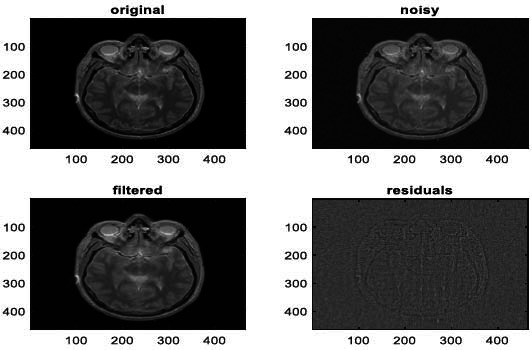
The fifth example of MRI denoising by applying the proposed image denoising technique.

**Fig. (15):**
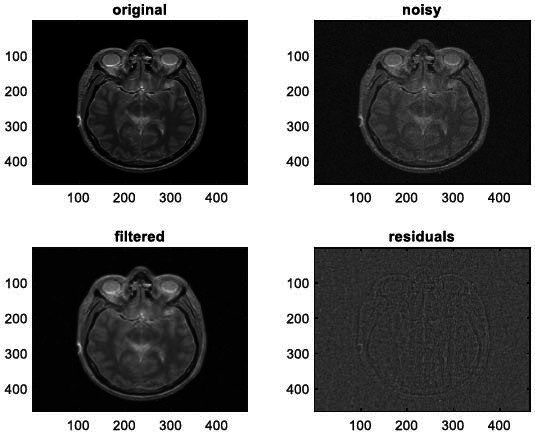
The sixth example of MRI denoising by applying the proposed image denoising technique.

**Fig. (16):**
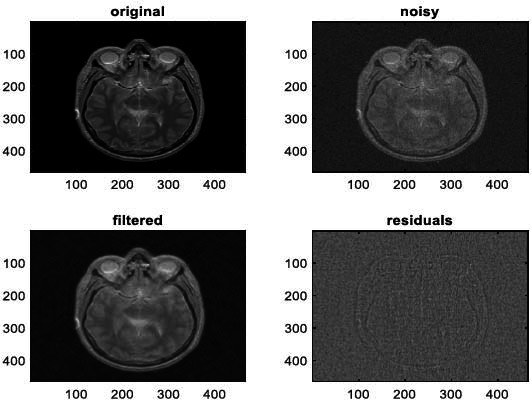
The seventh example of MRI denoising by applying the proposed image denoising technique.

**Fig. (17):**
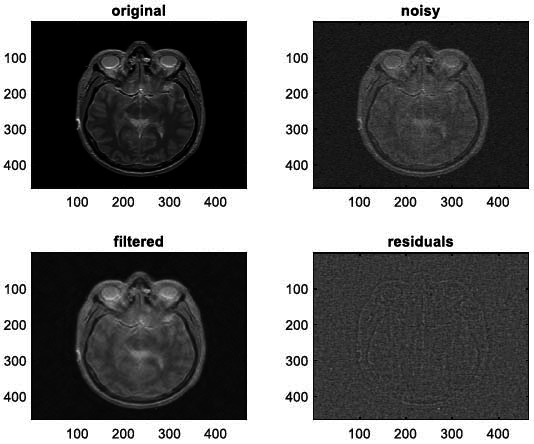
The eighth example of MRI denoising by applying the proposed image denoising technique.

**Fig. (18):**
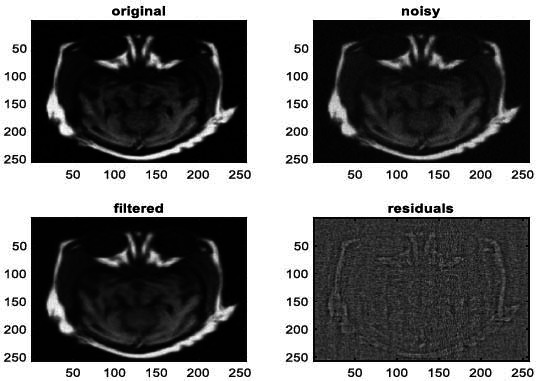
The ninth example of MRI denoising by applying the proposed image denoising technique.

**Fig. (19):**
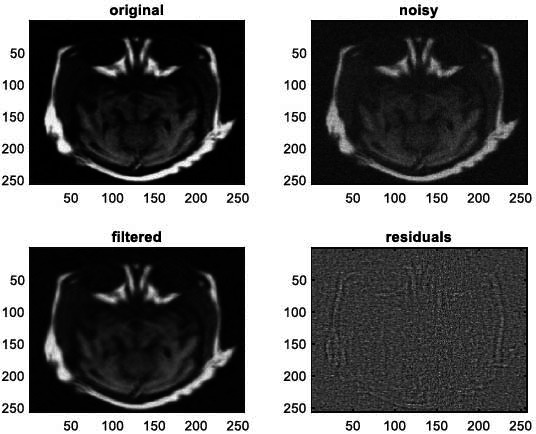
The tenth example of MRI denoising by applying the proposed image denoising technique.

**Fig. (20):**
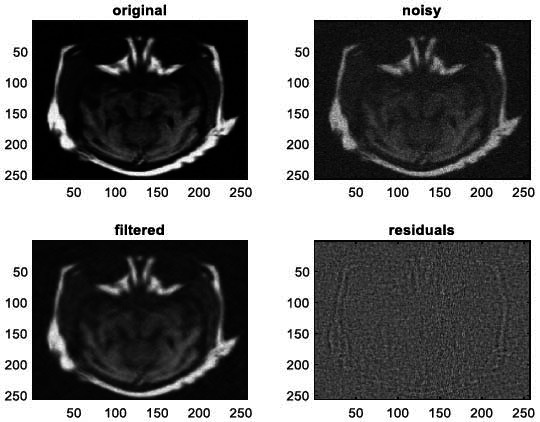
The eleventh example of MRI denoising by applying the proposed image denoising technique.

**Fig. (21):**
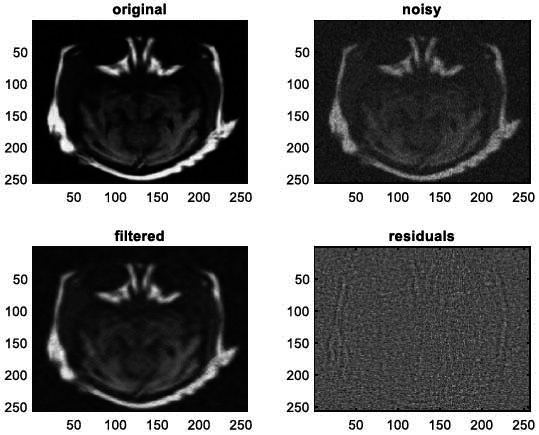
The twelfth example of MRI denoising by applying the proposed image denoising technique.

**Table 2:** The first example of MRI denoising: Clean Image ('input_MRI.jpg') corrupted by an Additive Gaussian White Noise (AGWN) with standard deviation, σ = 10.

**Sigma=10**	**The Proposed Technique**	**Non-local Means Filter [20]**
PSNR	**37.8076**	36.7189
SSIM	**0.9219**	0.8640
NMSE	**0.0869**	0.1375
FSIM	**0.9664**	0.9523

**Table 3:** The second example of MRI denoising: Clean Image ('input_MRI.jpg') corrupted by an Additive Gaussian White Noise (AGWN) with standard deviation, σ=20.

**Sigma=20**	**The Proposed Technique**	**Non-local Means Filter [20]**
PSNR	**33.5656**	31.4891
SSIM	**0.7884**	0.6434
NMSE	**0.0705**	0.1077
FSIM	**0.9339**	0.8883

**Table 4:** The third example of MRI denoising: Clean Image ('input_MRI.jpg') corrupted by an Additive Gaussian White Noise (AGWN) with standard deviation, σ=30.

**Sigma=30**	**The Proposed Technique**	**Non-local Means Filter [20]**
PSNR	**30.7345**	28.2278
SSIM	**0.6395**	0.4539
NMSE	**0.0725**	0.1227
FSIM	**0.8997**	0.8173

**Table 5:** The fourth example of MRI denoising: Clean image ('input_MRI.jpg') corrupted by an Additive Gaussian White Noise (AGWN) with standard deviation, σ=40.

**Sigma=40**	**The Proposed Technique**	**Non-local Means Filter [20]**
PSNR	**27.9419**	25.8125
SSIM	**0.4527**	0.3196
NMSE	**0.0856**	0.1522
FSIM	**0.8551**	0.7521

**Table 6:** The fifth example of MRI denoising: Clean -mage ('source18B.tif') corrupted by an addative Gaussian White Noise (AGWN) with standard deviation, σ=10.

**Sigma=10**	**The Proposed Technique**	**Non-local Means Filter [20]**
PSNR	34.6734	**34.9287**
SSIM	**0.8694**	0.8189
NMSE	**0.2019**	0.3301
FSIM	**0.9649**	0.9613

**Table 7:** The sixth example of MRI denoising: Clean image ('source18B.tif') corrupted by an Additive Gaussian White Noise (AGWN) with standard deviation, σ=20.

**Sigma=20**	**The Proposed Technique**	**Non-local Means Filter [20]**
PSNR	**30.507**	30.3047
SSIM	**0.6713**	0.5932
NMSE	**0.1823**	0.228
FSIM	**0.9213**	0.9071

**Table 8:** The seventh example of MRI denoising: Clean image ('source18B.tif') corrupted by an Additive Gaussian White Noise (AGWN) with standard deviation, σ = 30.

**Sigma=30**	**The Proposed Technique**	**Non-local Means Filter [** **20** **]**
PSNR	**27.2755**	27.2694
SSIM	**0.4620**	0.4358
NMSE	0.1649	**0.1575**
FSIM	**0.8701**	0.8481

**Table 9:** The eighth example of MRI denoising: Clean image ('source18B.tif') corrupted by an Addaiive Gaussian White Noise (AGWN) with standard deviation, σ=40.

**Sigma = 40**	**The Proposed Technique**	**Non local Means Filter [20]**
PSNR	**25.2564**	25.0927
SSIM	**0.3551**	0.3349
NMSE	**0.1787**	0.1822
FSIM	**0.8246**	0.7899

**Table 1:** Results in terms of PSNR and SSIM, obtained by applying the four denoising techniques [15-19].

**Noisy Image**	**The Denoising Technique**			
	**The Proposed Image Denoising Approach**	The Image Denoising Technique based on Thresholding in the SWT-2D domain [17]	**The Image Denoising Technique based on Deep Neural Network [** **19** **]**	**The Image Denoising Technique based on Soft Thresholding in the Domain of 2-D Dual-Tree Discrete Wavelet Transform [** **18** **]**
Noisy st.tif (σ = 10)	PSNR: **34.2517** SSIM: 0.8791	PSNR: 33.7831 SSIM: **0.8962**	PSNR: 28.3030 SSIM: 0.5425	PSNR: 34.1453 SSIM: 0.8675
Noisy st.tif (σ = 20)	PSNR: **30.6126** SSIM: **0.7722**	PSNR: 30.1884 SSIM: 0.6950	PSNR: 22.4725 SSIM: 0.2850	PSNR: 30.2840 SSIM: 0.7306
Noisy st.tif (σ = 30)	PSNR: **28.1244** SSIM: **0.6131**	PSNR: 24.5706 SSIM: 0.3719	PSNR: 18.9533 SSIM: 0.1789	PSNR: 27.7900 SSIM: 0.5977
Noisy st.tif (σ = 40)	PSNR: **26.4861** SSIM: **0.5138**	PSNR: 20.6093 SSIM: 0.2171	PSNR: 16.4559 SSIM: 0.1248	PSNR: 25.9906 SSIM: 0.4953
Noisy Peppers..tif (σ = 10)	PSNR: **32.8048** SSIM: **0.8250**	PSNR: 32.4622 SSIM: 0.8019	PSNR: 28.6273 SSIM: 0.6497	PSNR: 32.6519 SSIM: 0.8180
Noisy Peppers.tif (σ = 20)	PSNR: **29.6501** SSIM: **0.7233**	PSNR: 29.3674 SSIM: 0.6821	PSNR: 22.4648 SSIM: 0.3569	PSNR: 29.5645 SSIM: 0.7038
Noisy Peppers.tif (σ = 30)	PSNR: **27.8670** SSIM: **0.6406**	PSNR: 27.8481 SSIM: 0.6264	PSNR: 18.8973 SSIM: 0.2208	PSNR: 27.4151 SSIM: 0.5968
Noisy Peppers.tif (σ = 40)	PSNR: **26.3256** SSIM: **0.5462**	PSNR: 26.2747 SSIM: 0.5424	PSNR: 16.3708 SSIM: 0.1497	PSNR: 25.7770 SSIM: 0.5109
Noisy House.tif (σ = 10)	PSNR: **35.0397** SSIM: **0.9203**	PSNR: 34.2284 SSIM: 0.8466	PSNR: 28.4578 SSIM: 0.5576	PSNR: 35.0360 SSIM: 0.9121
Noisy House.tif (σ = 20)	PSNR: **30.7882** SSIM: **0.8462**	PSNR: 30.3082 SSIM: 0.7360	PSNR: 22.4921 SSIM: 0.3090	PSNR: 30.5116 SSIM: 0.7766
Noisy House.tif (σ = 30)	PSNR: **28.1921** SSIM: **0.6586**	PSNR: 27.0232 SSIM: 0.5584	PSNR: 18.9670 SSIM: 0.2049	PSNR: 27.9388 SSIM: 0.6487
Noisy House.tif (σ = 40)	PSNR: **26.2186** SSIM: 0.5368	PSNR: 25.4000 SSIM: 0.4792	PSNR: 16.4148 SSIM: 0.1455	PSNR: 26.0214 SSIM: **0.5416**

**Table 10:** The ninth example of MRI denoising: Clean image ('med256B.jpg') corrupted by an Additive Gaussian White Noise (AGWN) with standard deviation, σ=10.

**Sigma=10**	**The Proposed Technique**	**Non-local Means Filter [20]**
PSNR	34.1486	**34.9727**
SSIM	**0.9026**	0.8981
NMSE	0.2179	**0.191**
FSIM	0.9542	**0.9579**

**Table 11:** The tenth example of MRI denoising: Clean image ('med256B.jpg') corrupted by an Additive Gaussian White Noise (AGWN) with standard deviation, σ=20.

**Sigma=20**	**The Proposed Technique**	**Non-local Means Filter [20]**
PSNR	**30.7208**	30.4004
SSIM	**0.7978**	0.7559
NMSE	**0.1711**	0.1762
FSIM	**0.9159**	0.9152

**Table 12:** The eleventh example of MRI denoising: Clean image ('med256B.jpg') corrupted by an Additive Gaussian White Noise (AGWN) with standard deviation, σ=30.

**Sigma=30**	**The Proposed Technique**	**Non-local Means Filter [20]**
PSNR	**28.0427**	27.1948
SSIM	**0.6728**	0.6146
NMSE	**0.1752**	0.1941
FSIM	**0.8782**	0.8664

**Table 13:** The twelfth example of MRI denoising: Clean image ('med256B.jpg') corrupted by an Additive Gaussian White Noise (AGWN) with standard deviation, σ=40.

**Sigma=40**	**The Proposed Technique**	**Non-local Means Filter [20]**
PSNR	**26.467**	25.0481
SSIM	**0.6036**	0.5121
NMSE	**0.1505**	0.2163
FSIM	**0.845**	0.8203

## Data Availability

All data generated or analyzed during this study are included in this published article.

## LIST OF ABBREVIATIONS

AGWN: Additive Gaussian White Noise
db4: Daubechies four
DWT: Discrete Wavelet Transform
DT-CWT: Dual Tree Complex Wavelet Transform
FSIM: Feature Similarity Index
I_b_: Noisy Image
I_d_: Denoised Image
MRIs: Magnetic Resonance Images
NLM: Non-Local Means
NMSE: Normalized Mean Square Error
σ: Standard Deviation (te noise level)
PSNR: Peak Signal-to-Noise Ratio
SSIM: Structural Similarity Index
SWT-2D: Two-Dimensional Stationary Wavelet Transform
SWT_-1_-2D: Inverse Two-Dimentional Stationary Wavelet Transform
Wb{j}{s},1 ≤ j ≤ 2, 1 ≤ s ≤ 3: Noisy Wavelet Coefficients
Wd{j}{s},1 ≤ j ≤ 2, 1 ≤ s ≤ 3: Denoised Wavelet Coefficients
